# Transition of dislocation nucleation induced by local stress concentration in nanotwinned copper

**DOI:** 10.1038/ncomms8648

**Published:** 2015-07-16

**Authors:** N. Lu, K. Du, L. Lu, H. Q. Ye

**Affiliations:** 1 Shenyang National Laboratory for Materials Science, Institute of Metal Research, Chinese Academy of Sciences, Shenyang 110016, China.

## Abstract

Metals with a high density of nanometre-scale twins have demonstrated simultaneous high strength and good ductility, attributed to the interaction between lattice dislocations and twin boundaries. Maximum strength was observed at a critical twin lamella spacing (∼15 nm) by mechanical testing; hence, an explanation of how twin lamella spacing influences dislocation behaviours is desired. Here, we report a transition of dislocation nucleation from steps on the twin boundaries to twin boundary/grain boundary junctions at a critical twin lamella spacing (12–37 nm), observed with *in situ* transmission electron microscopy. The local stress concentrations vary significantly with twin lamella spacing, thus resulting in a critical twin lamella spacing (∼18 nm) for the transition of dislocation nucleation. This agrees quantitatively with the mechanical test. These results demonstrate that by quantitatively analysing local stress concentrations, a direct relationship can be resolved between the microscopic dislocation activities and macroscopic mechanical properties of nanotwinned metals.

## Abstract (web summary)

Metallic materials with a nanometre-scaled lamella structure can have properties that are very different from their coarser-grained counterparts. Here, the authors demonstrate how dislocations in such a material—nanotwinned copper—can nucleate in two distinctly different mechanisms depending on local stress

## Graphical abstract

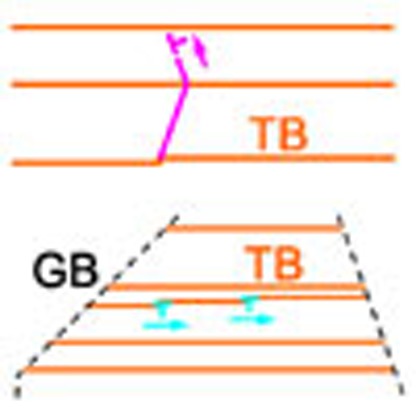

Materials with nanometre-scale twins have attracted intensive research interest, attributed to their potential for achieving extraordinary properties compared with their coarsen-grained counterparts. For instance, nanotwinned metals exhibit high strength, good ductility and promising electric conductivity^1^,^2^. The mechanical properties are associated with interactions between lattice dislocations and the coherent twin boundaries (TBs), as TBs serve as strong barriers for the motion of dislocations^1^,^3^,^4^,^5^,^6^,^7^. Accordingly, the strength of nanotwinned metals supposedly increases with decreasing twin lamella spacing, as is the case when the twin lamella spacing decreases from hundreds down to tens of nanometres^8^. Nevertheless, when twin lamella spacing decreases below a critical thickness, ∼15 nm for nanotwinned copper, a softening behaviour instead of strengthening prevails for the tensile strength; therefore, the yield stress decreases with further reduced twin lamella spacing^8^.

The dislocation activities in nanotwinned metals can be divided into three types^9^,^10^,^11^: Hard mode I, dislocations slip along the {111} planes that cross the twin lamellae; Hard mode II, threading dislocations traverse through the twin lamellae where the slip planes are inclined to TBs; and Soft mode, dislocations initiate from TB/GB (grain boundary) junctions and then slip along the coherent TBs. Molecular dynamics (MD) simulations^12^ suggested that the softening corresponds to the dominant activity of Soft mode dislocations below a critical twin lamella spacing, where the dislocations nucleate from the TB/GB intersections. A critical spacing of 2–3 nm was determined for the mean grain size of 20 nm by a three-dimensional (3D) MD simulation. The critical twin lamella spacing depends on the mean size of the grains: the larger the mean grain size is, the larger the critical lamella spacing. Other MD simulations^13^ suggested that a transition of Lomer dislocation behaviours occurring at a critical twin spacing of 2.4 nm causes the transition from strengthening to softening. Zhou *et al*.^14^ recently revealed that a transition from the activation of threading dislocations to the collective motion of multiple extended jogged dislocations governed the deformation mechanism in columnar-grained nanotwinned metals, where the presence of TBs always strengthens the metals. Wang *et al*.^4^ proposed that partial dislocations can glide along the lines of steps with a height of multiple layers, leading to softening in the columnar-grained nanotwinned Cu. Hence, experimental investigations are desirable to resolve the actual deformation mechanisms in nanotwinned metals and their relationship with the mechanical properties.

Here, we have investigated an equiaxial-grained nanotwinned Cu with a combination of *in situ* high-resolution transmission electron microscopy (HRTEM) and quantitative strain analysis. The mean grain size of the nanotwinned Cu used in our experiment is 400–600 nm. The transition of two types of dislocation activities is revealed at a critical twin lamella spacing (12–37 nm), where type I dislocations (Hard mode I) are emitted from steps on TBs and glided on {111} planes inclined to the TBs, while type III dislocations (Soft mode) are emitted from TB/GB junctions and slip on the TBs. This competition is governed by the stress concentration at the steps on TBs and TB/GB junctions. In addition, a strong dependence of twin lamella spacing on the local stress concentration is detected; thus, a critical twin lamella spacing is determined as ∼18 nm for the transition from strengthening to softening in nanotwinned Cu. The result agrees quantitatively with the experimentally measured critical spacing (15 nm). These data provide insights into the direct relationship between the local dislocation activities and macroscopic mechanical properties of bulk materials with geometrically designed nanostructures such as nanotwins.

## Results

### Dislocation activities during incipient deformation process

**Figure 1:**
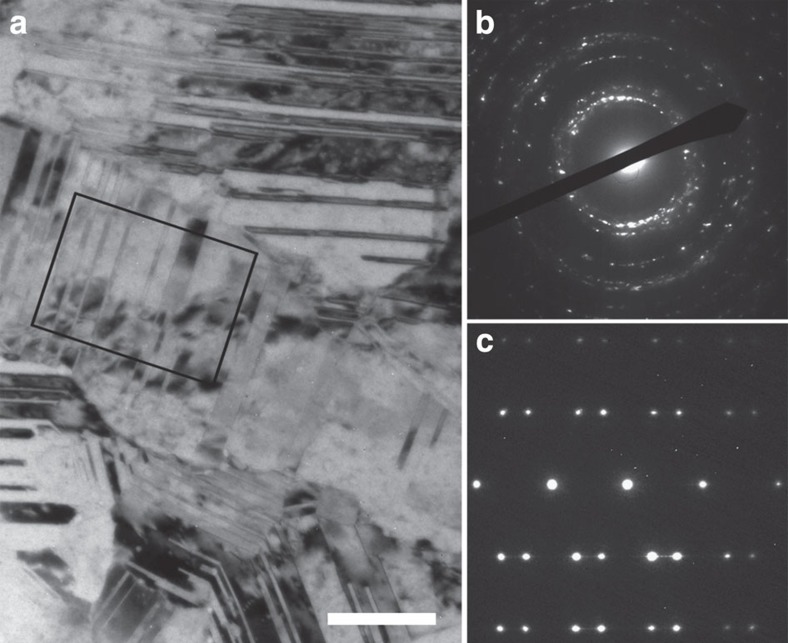
Microstructure of as-deposited Cu with nanoscaled twins. (**a**) Bright-field TEM image. Scale bar, 50 nm. (**b**) Select-area electron diffraction pattern of the observed area in **a**. (**c**) Diffraction pattern obtained from a smaller area, indicated by a black box in **a**.

**Figure 2:**
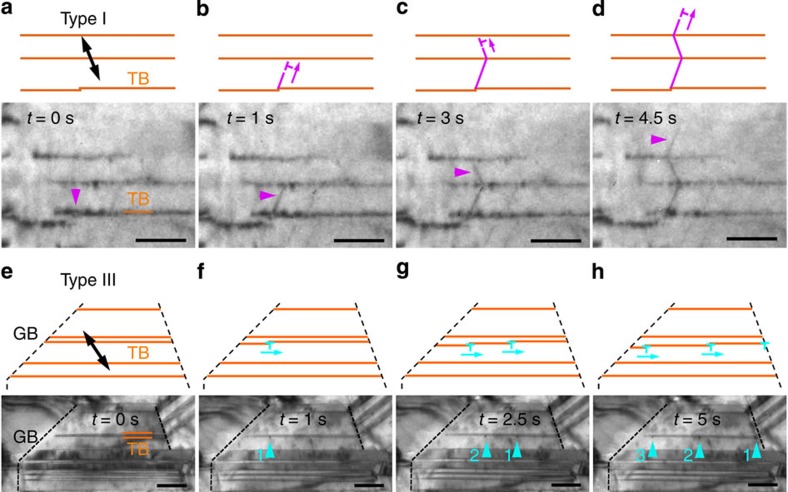
*In situ* bright-field TEM images under tensile stress. (**a**–**d**) Dynamic process of a dislocation nucleated from a step on a coherent TB and then gliding on a {111} plane inclined to the TB. The dislocation is indicated by a magenta arrow. Scale bars, 40 nm. (**e**–**h**) Dynamic process of a sequence of dislocations emitted from a TB/GB junction and then gliding along the TB. The dislocations are indicated by blue arrows 1–3. Scale bars, 50 nm. Corresponding schematics are above the bright-field images. The loading direction is indicated by double-headed arrows.

The studied sample here is an equiaxial-grained nanotwinned Cu (ref. 15), as shown in Fig. 1. *In situ* TEM observation (Fig. 2) reveals mainly two types of dislocation activities during the tensile loading on the nanotwinned copper (see Supplementary Table 1): type I (Hard mode I) and type III (Soft mode) dislocations.

Time-resolved bright-field images and corresponding schematics (Fig. 2a–d) show the dynamic process of type I dislocations slipping on a plane inclined to the coherent TB. The main defects in as-deposited nanotwinned copper are steps having a height of no more than two {111} layers on the TBs. Two types of steps are observed as glissile and sessile ones (for details, see the Supplementary Note 1). Figure 2a shows a step, presumably a sessile one, located at a coherent TB. Under the applied stress, a dislocation was emitted from the step and further passed through the twin lamellae (Fig. 2b–d). Detailed information can be seen in Supplementary Movie 1. Other examples of the emission of type I dislocations are also presented in Supplementary Fig. 1. Statistical results on steps with dislocation emission by post-mortem HRTEM also suggest that steps with heights of no more than 2 {111} layers are the major sites (more than 89%) for type I dislocation emission. This suggests that steps with 1 or 2 {111} layers play a dominant role in the emission of type I dislocations in the incipient deformation (for details, see the Supplementary Note 2).

Figure 2e–h illustrates type III dislocations slipping along coherent TB planes. At the beginning, the coherent TBs are smooth and clean with no dislocations visible on them (for example, Fig. 2e). With the loading of tensile stress, a sequence of dislocations, indicated by blue arrows, was observed being emitted from the TB/GB junction and, subsequently, gliding along the TB (Fig. 2f–h). Detailed information can be seen in Supplementary Movie 2. Supplementary Fig. 2 also presents other examples of the emission of type III dislocations.

### Proportions of two types of dislocations

**Figure 3:**
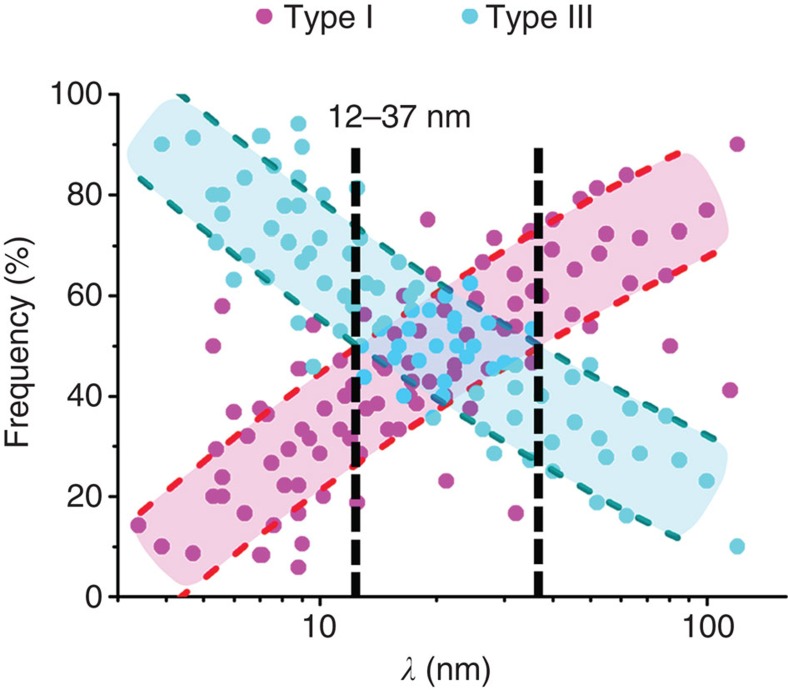
Statistical distribution of two types of dislocations in nanotwinned Cu with different twin lamella spacings *λ* during *in situ* deformations. Proportions of type I and III dislocations are indicated by magenta and blue symbols, respectively. The fitting lines are determined by polynomial fitting of the experimental data, while the bounding lines mark the s.d. of the data points.

Figure 3 is a statistical result on the proportion of each type of dislocation for different twin lamella spacings (hereafter referred to as *λ*) from TEM observations. Here the total numbers of samples of active dislocations and twin lamellae are more than 1,500 and 100, respectively. Every set of data points indicates the proportions of type I and type III dislocations in one twin lamella. For example, when the numbers of type I and type III dislocations in a lamella are 9 and 14, the proportions of type I and III dislocations are 39% and 61%, respectively. From this result, a competition is revealed between type I and III dislocations. The proportion of type I dislocations decreases with decreasing *λ* from 65 to 3 nm. This is in contrast to type III dislocations, which increase significantly with decreasing *λ*. For *λ*>37 nm, dislocation slips across twin lamellae that is, type I dislocations, are the majority. For *λ*<12 nm, the proportion of type III dislocations becomes greater than 50%, which suggests that dislocation slips along the twin lamella direction dominate the plastic deformation. Accordingly, a critical twin lamella spacing *λ*_c_ of 12–37 nm is determined for the transition of major dislocation activities. This is quantitatively consistent with the value of *λ*_c_ (15 nm) between the strengthening and softening of the nanotwinned Cu determined by mechanical tests^8^.

### Stress concentration at steps on TBs and TB/GB junctions

**Figure 4:**
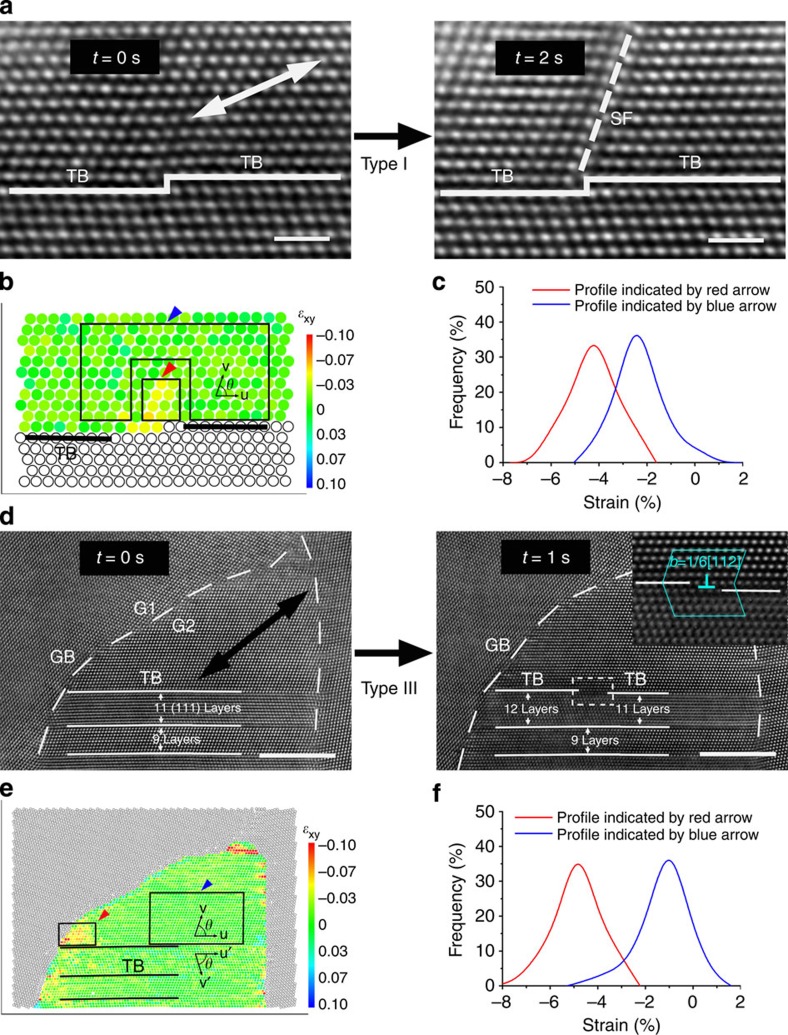
*In situ* HRTEM images during tensile loading. (**a**) Dynamic process of type I dislocations emitted from a step on the TB. Scale bars, 1 nm. (**b**) The lattice shear strain determined before the emission, corresponding to the left image of **a**. (**c**) Quantitative shear strain analysis of the black-box regions indicated by red and blue arrows in **b**. (**d**) Dynamic process of type III dislocations emitted from a TB/GB junction and then slipping along the TB. G1 and G2 are two grains. Scale bars, 5 nm. (**e**) The lattice shear strain determined before the emission (the left image of **d**). (**f**) Quantitative shear strain analysis of the black-box regions indicated by red and blue arrows in **e**. The loading direction is indicated by double-headed arrows.

The dynamical process of dislocations being emitted from steps on coherent TBs was captured with an *in situ* HRTEM investigation (for example, Fig. 4a). As shown in the left image of Fig. 4a, a sessile step was observed on the TB (see details in the Supplementary Note 3). After applying tensile stress, a partial dislocation was emitted from the step and glided into the twin lamella along a {111} plane inclined to the TB, leaving behind a stacking fault (the right image of Fig. 4a). The location of the step is determined from two referenced planes, which are a coherent TB at 28 (111) layers beneath the step and an immobile incoherent TB on the right (see Supplementary Fig. 3). In this way, the step is verified as remaining in the same position during the entire dislocation emission process.

*In situ* HRTEM was also conducted to observe the dislocation emission from a TB/GB junction (Fig. 4d). Here the misorientation angle *α* is 35.3° between grain 1 (G1) and grain 2 (G2), corresponding to a high angle GB. The twin lamella spacing *λ* is ∼2 nm in grain 2. TBs were smooth without any visible defects before the dislocation emission (the left image of Fig. 4d). After applying tensile stress, a dislocation (marked by a box and enlarged in the inset) was observed being emitted from the left TB/GB junction (the right image of Fig. 4d). By applying the Frank circuit approach^16^,^17^, the dislocation was identified as a 30° Shockley partial dislocation with a Burgers vector of *a*/6[112].

To determine the stress state for the dislocation emission at the steps, a quantitative analysis was conducted with the LADIA programme^18^,^19^,^20^ on the time-resolved HRTEM images. The shear stress before the dislocation emission (the left image of Fig. 4a) is shown in Fig. 4b, where a significant stress concentration is discovered in the vicinity of the steps. Quantitative analysis (Fig. 4c) on strain gauges marked by black boxes (indicated by red and blue arrows) in Fig. 4b indicates that the mean shear strains near the step and in the surrounding grain interior, *ɛ*_local_^I^ and *ɛ*_global_^I^, are 0.039±0.003 and 0.023±0.001 (all the error bars here represent the s.e.), respectively. The validity of the selected strain gauge around the step is discussed in the Supplementary Note 4. The above results suggest that a critical shear stress *τ*_critical_^I^ for the emission of type I dislocations is ∼1.87±0.14 GPa (given that the shear modulus is 48 GPa on {111} planes for Cu (ref. 21)). The critical shear stresses as estimated from other two examples (1.69±0.10 and 1.93±0.05 GPa) are consistent with this value (see details in Supplementary Fig. 4). On the basis of the local shear stress near the step and in the grain interior, a stress concentration factor *K* is estimated as *K*_I_=*ɛ*_local_^I^/*ɛ*_global_^I^=1.70±0.21 for the steps on TBs, where I stands for the type I dislocations. In addition, the influence of step height on the critical stress required for the emission of type I dislocations has been discussed in the Supplementary Note 5.

The stress state before the dislocation emission (the left image of Fig. 4d) at the TB/GB junction was also resolved with the quantitative strain analysis (LADIA programme) on *in situ* HRTEM images (Fig. 4e,f). According to the same procedure as shown in Fig. 4b,c, quantitative analysis (Fig. 4f) on strain gauges marked by black boxes (indicated by red and blue arrows) in Fig. 4e indicates that the mean shear strains at the TB/GB junction and of the interior grain (referred to as *ɛ*_local_^III^ and *ɛ*_global_^III^) are 0.045±0.002 and 0.011±0.001, respectively. These results suggest that the critical shear stress *τ*_critical_^III^ corresponding to the dislocation emission is ∼2.16±0.10 GPa. The shear strain after the dislocation emission (*t*=1 s) is 0.042±0.002, which is roughly the same as the strain before the dislocation emission (see details in the Supplementary Note 6). This is probably the reason that dislocations are continuously emitted from TB/GB junctions, as shown in Fig. 2. Therefore, a stress concentration factor at the TB/GB junction is estimated as *K*_III_=*ɛ*_local_^III^/*ɛ*_global_^III^=4.10±0.60. The influence of grain boundary structures and twin lamella spacing on the critical stress required for the nucleation of type III dislocations is investigated in the Supplementary Note 7.

### Stress concentration at various twin lamella spacings

**Figure 5:**
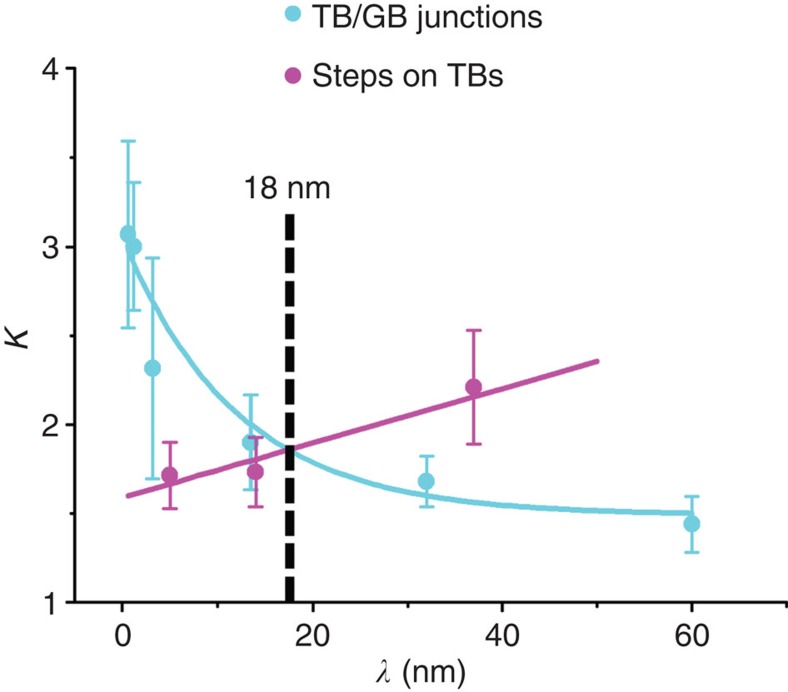
The stress concentration factors in nanotwinned Cu with different twin lamella spacings. The stress concentration factors *K* measured at steps on TBs and TB/GB junctions for nanotwinned Cu with different TB spacings *λ*. The data are plotted and indicated by magenta and blue solid circles.

According to the same method as shown in Fig. 4, the stress concentration factors *K* at steps on TBs and TB/GB junctions are calculated for different *λ* values, as shown in Fig. 5. Examples of these measurements can also be seen in Supplementary Figs 4 and 5. With the decrease in *λ*, the stress concentration at steps on TBs decreases slightly. On the contrary, the stress concentration at TB/GB junctions increases significantly with decreasing twin lamella spacing; therefore, for the same applied global stress, higher local shear stress will be induced at TB/GB junctions for twin lamellae with smaller *λ*. The two *K*∼*λ* curves for type I and III dislocations crossover at *λ*≈18 nm, which agrees well with the above result of the *λ*_c_ (12–37 nm) in Fig. 3. Here the stress concentration factors at TB/GB junctions are all determined at near Σ9 GBs because Σ9 GB has the highest proportion among all the GBs and is favourable for the nucleation of type III dislocations (see details in the Supplementary Note 7).

## Discussion

Because the above dislocation behaviours are governed by dislocation nucleation and are thus related to local stress concentration, the local stress needed for dislocation nucleation predicts the dominant dislocation mechanism for a given *λ* value. For a larger *λ*>*λ*_c_, type I dislocations would dominate the deformation process. Correspondingly, the local stress at steps on TBs should be greater than the stress at TB/GB junctions, that is,


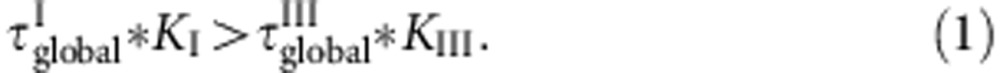


The sample used in the experiment is equiaxial-grained Cu with no observable preferred orientation in the foil plane. Therefore, for a random individual grain, the resolved shear stress could be assumed to be the same for slip systems of type I or type III dislocations from a statistical view. For the effect of stress concentration, the resolved shear stress induced by the applied external stress would correspond to the global stress. In this way, *τ*_global_^I^ is assumed to be the same as *τ*_global_^III^ from a statistical view, for simplicity. Thus, equation (1) can be simplified as


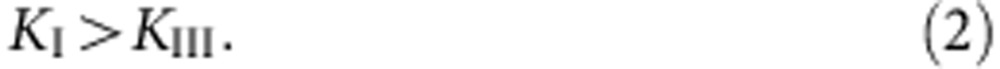


Similarly, *K*_I_<*K*_III_ is applied for the regime where type III dislocations dominate at smaller *λ* values, *λ*<*λ*_c_, as well as *K*_I_=*K*_III_ for *λ*_c_. Therefore, the local stress concentration effect is critical to define the dominant dislocation activity and the critical twin lamella spacing. This effect would explain the fact that the variations of stress concentration factors with *λ* (Fig. 5) could lead to the corresponding critical *λ* of ∼18 nm, when the global shear stress is assumed as the same for different slip systems from the perspective of statistics. The critical twin lamella spacing (∼18 nm) agrees quantitatively well with the result (∼15 nm) determined from the experimental mechanical tests.

**Figure 6:**
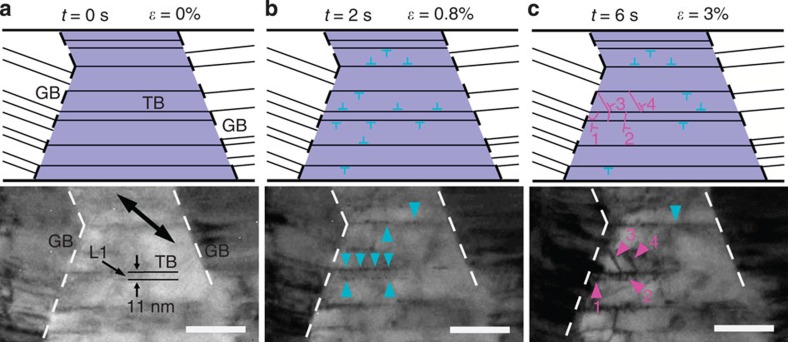
Dynamic process under a large deformation strain in the nanotwinned Cu. (**a**–**c**) Time-resolved bright-field TEM images and corresponding schematic illustrations recorded for the apparent shear strain values of 0, 0.8 and 3%. Dislocations gliding along TBs or planes inclined to TBs are indicated by blue or magenta arrows, respectively. Dislocation 1 appears as a half-loop shape. Dislocations 2, 3 and 4 appear as straight lines. The corresponding movie is given in Supplementary Movie 3. Scale bars, 100 nm. The loading direction is indicated by double-headed arrows.

For the strain-hardening process, the post-mortem experiment in ref. 8 shows that abundant dislocations across twin lamellae (that is, not type III dislocations) are blocked by TBs even for very small twin lamella spacings (4 nm) at a large strain of 30%. Meanwhile, the MD simulations^12^ show that the deformation is still mainly governed by the activities of type III dislocations at a strain of 10%, where strain hardening is not observed. Therefore, to quantitatively resolve the dislocation activities during the deformation, it is necessary to estimate the valid range of the incipient deformation process and to investigate the onset of the strain-hardening effect. The deformation process under a large strain (∼3%) in nanotwinned Cu is revealed in time-resolved TEM images and corresponding schematics (Fig. 6a–c). Here the *λ* (∼11 nm) of twin lamella 1 (indicated as L1) is less than the critical *λ*_c_, as shown in Fig. 6a. Initially, there are rare dislocations within the nanotwinned Cu (Fig. 6a). After the loading of external stress, abundant type III dislocations (indicated by blue arrows) nucleate from TB/GB junctions and then glide along the TBs, as shown in Fig. 6b. With the apparent shear strain increasing to 3% (Supplementary Note 8), type I dislocations (indicated by magenta arrows 1 and 2) are emitted from TB/GB junctions or steps on TBs and then slip across twin lamellae on 
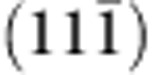
 planes inclined to the TBs (Fig. 6c), corresponding to the strain-hardening effect. The mechanism of deformation with a large strain here is different from the process described above with regard to the incipient deformation (for details, see the Supplementary Note 9). This result is also consistent with the phenomenon of dislocations being blocked at TBs for *λ*=4 nm in the post-mortem experiment of the 30% deformed sample^8^. Therefore, the adoption of the range of incipient deformation as ∼1% is reasonable for the statistics on the proportions of different types of dislocations.

In summary, from the statistics of dislocation activities using *in situ* TEM, the quantitative analysis of the local stress concentration from time-resolved HRTEM and the result from the mechanical test^8^, a direct relationship can be established between the dislocation activities and mechanical properties of nanotwinned metals. In a broader view, the *in situ* TEM and the quantitative analysis of lattice distortion may provide insight into connecting the local dislocation behaviours and predicted mechanical properties of bulk materials with geometrically designed nanostructures.

## Methods

### Sample preparation and TEM characterization

Equiaxial-grained nanotwinned Cu foil samples were synthesized by means of pulsed electrodeposition^8^. The as-deposited Cu foils have dimensions of 20 mm × 10 mm × 50 μm.

TEM specimens were thinned with double-jet electropolishing in a solution of 25% phosphoric acid, 25% ethanol and 50% distilled water at 263 K. TEM observations were conducted using a FEI Tecnai F30 electron microscope operating at 300 kV with a spatial resolution of 0.20 nm and a FEI Titan aberration-corrected electron microscope operating at 300 kV with a spatial resolution of 0.08 nm. An *in situ* TEM investigation was conducted using a Gatan model 654 single-tilt straining holder at room temperature^19^,^22^. Time-resolved bright-field TEM and HRTEM images were taken during the intermittent straining. During the *in situ* tensile experiment, dislocation activities are normally observed under the loading with a tensile displacement of up to 15–20 μm. The thin area of the sample, where most of the tensile stress is loaded and is thus presumably the deformed area under the tensile loading, is ∼2–2.5 mm in length (Supplementary Fig. 18). Therefore, the apparent strain can be roughly estimated by the ratio of the tensile displacement to the length of the deformed area as ∼1%.

### Strain analysis by LADIA

The LADIA programme^18^ was used to analyse the stress state for dislocation emissions during tensile deformation. The angle *θ* between two vectors **u** (1/4
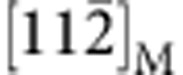
 in the (111)_M_ plane) and **v** (
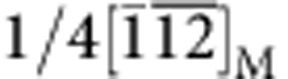
 in the 
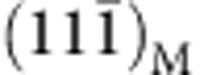
 plane) is employed to estimate the lattice shear strain of the matrix. The counterpart basic vectors in twin lamellae are **u′** 
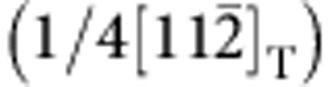
 and **v′** 
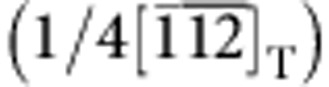
 (Fig. 4b,e). The undistorted angle *θ*_0_ is 70.53° for a face-centred cubic crystal. Under a shear stress, the angle *θ* will vary with the lattice distortion. The stress concentration factor *K* is estimated by the ratio of the local shear strain for dislocation nucleation and the global strain in the grain interior.

On the accuracy of quantitative analyses on local stress states, we observe that for Fig. 4a the image features and contrast remain constant throughout the observation area before and after the dislocation emission, which indicates that the focus setting and the height of the specimen remain steady and there is no significant bending or twisting of the sample in the observation area during the dislocation emission. Similarly, for Fig. 4d, the image features and contrast are nearly the same before and after the dislocation emission, which also suggests that the focus setting and the height of the specimen remain the same. According to our previous simulation^23^, slight bending or twisting of the crystal will not introduce significant effects on the accuracy of the lattice distortion measurement. Statistics on the strain gauges will further decrease the influence of local variations in the imaging parameters and delocalization effects on the accuracy of quantitative analysis. Therefore, it is reliable to make the comparison of the averaged strains determined from the strain gauges.

## Additional information

**How to cite this article:** Lu, N. *et al*. Transition of dislocation nucleation induced by local stress concentration in nanotwinned copper. *Nat. Commun.* 6:7648 doi: 10.1038/ncomms8648 (2015).

## Supplementary Material

Supplementary Figures, Supplementary Tables, Supplementary Notes and Supplementary References Supplementary Figures 1-18, Supplementary Tables 1-2, Supplementary Notes 1-9 and Supplementary References

Supplementary Movie 1 Dynamic process of a dislocation nucleated from a step on a coherent twin boundary (TB) and then glided on a {111} plane inclined to the TB.

Supplementary Movie 2 Dynamic process of a sequence of dislocations emitted from a twin boundary/grain boundary junction and then glided along the TB.

Supplementary Movie 3 Dynamic process under a large deformation strain in the nanotwinned Cu.

Supplementary Movie 4 Dynamic process of dislocations nucleated from steps on TBs and then glided on {111} planes inclined to the TBs.
