# Supplementary material for: Transition of dislocation nucleation induced by local stress concentration in nanotwinned copper
Source: Nat Commun. 2015 Jul 16;6:7648. doi: 10.1038/ncomms8648 (PMC4518316; doi:10.1038/ncomms8648)
Supplement: Supplementary Figures, Supplementary Tables, Supplementary Notes and Supplementary References — Supplementary Figures 1-18, Supplementary Tables 1-2, Supplementary Notes 1-9 and Supplementary References [file ncomms8648-s1.pdf]

Supplementary Figures

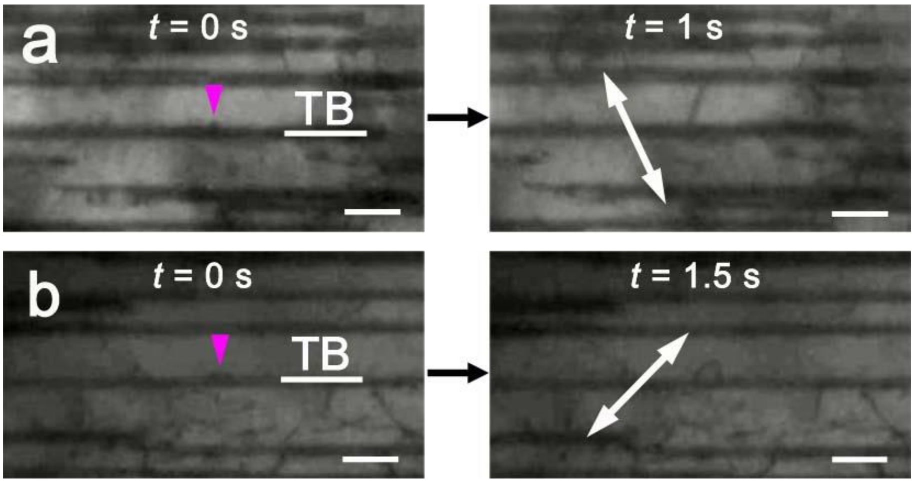

Supplementary Figure 1: More examples of the emission of type I dislocations. (a) A type I dislocation was emitted from a presumably sessile step on a twin boundary (TB) and slipped along a {111} plane inclined to the TB. The dislocation appears as a straight line, because its slipping plane is edge-on in the viewing direction. **(b)** Another type I dislocation was emitted from a TB and slipped on a plane inclined to the twin boundary. The half-loop shape of the dislocation indicates that its slipping plane is inclined to the viewing direction. Type I dislocations are indicated by magenta arrows. The loading direction is indicated by double headed arrows. All the scale bars represent 100 nm.

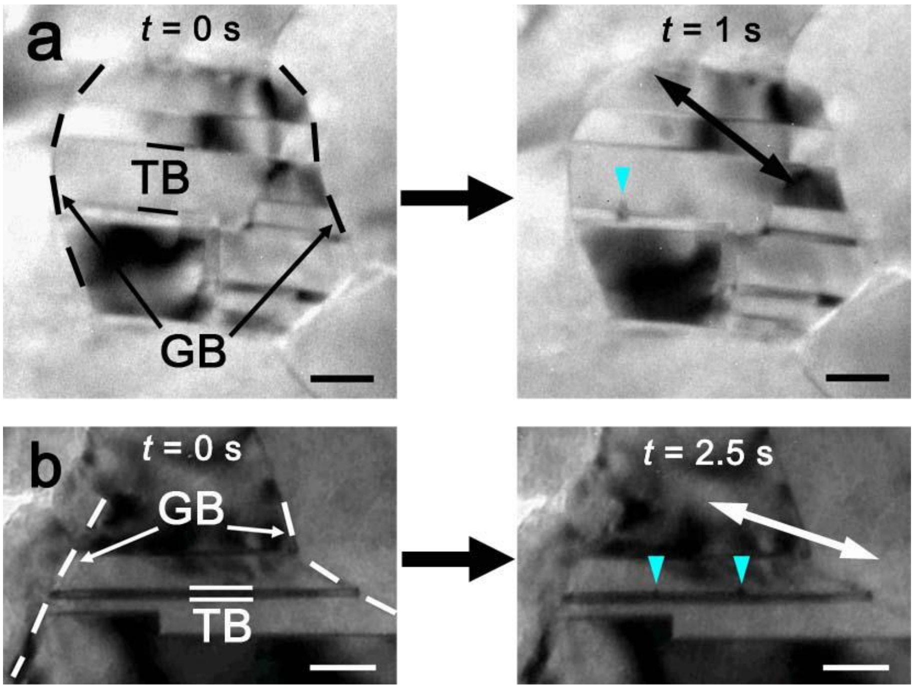

Supplementary Figure 2: More examples of the emission of type III dislocations. **(a)** A type III dislocation slips on the twin boundary. The dislocation was presumably emitted from the left twin boundary/grain boundary (TB/GB) junction. The scale bar represents 20 nm. **(b)** Two type III dislocations were sequentially emitted from a TB/GB junction and slipped on the twin boundary. The type III dislocations are indicated by blue arrows. The scale bar represents 10 nm.The loading direction is indicated by double headed arrows.

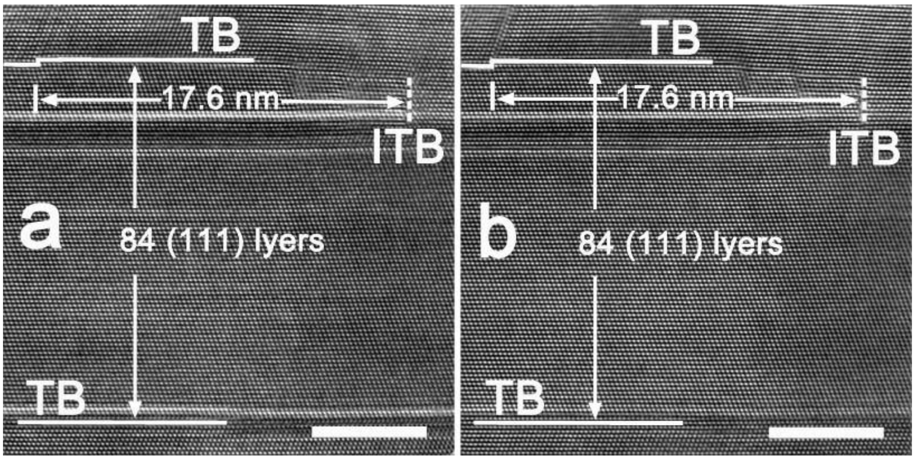

Supplementary Figure 3: Location of the step on the TB before (a) and after (b) the dislocation emission. The images covering the larger area are shown in **(a)** and **(b)** corresponding to the left and right images of Fig. 4a in the main text. Two reference lines, which are a coherent twin boundary at 84 (111) layers beneath the twin boundary and an immobile incoherent twin boundary (ITB) on the right, remain in the same place before and after the emission of a dislocation. Scale bar: 5 nm.

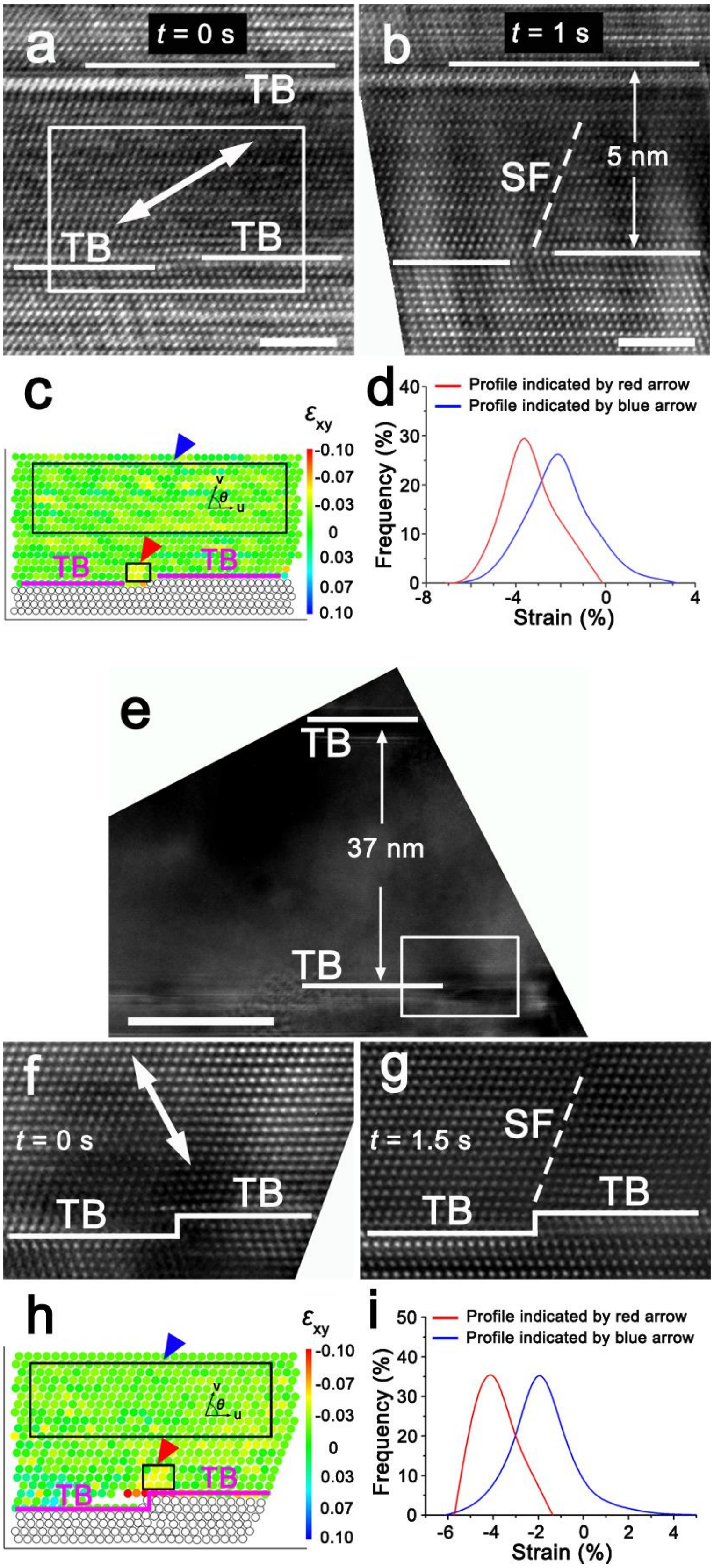

Supplementary Figure 4: The stress state for the emission of dislocations from steps on twin boundaries. (a-b) Sequential HRTEM images showing the emission of

a dislocation from a step on a TB. The thickness of the twin lamella is approximately 5 nm. Scale bars: 2 nm. **(c)** The lattice shear strain determined before emission, corresponding to **(a)**. **(d)** Quantitative shear strain analysis of the black-box regions indicated by red and blue arrows in **(c)**. The mean shear strains near the step and in the surrounding grain interior, ε_{local}^{I} and ε_{global}^{I}, are 0.035 ± 0.002 and 0.020 ± 0.001, respectively. **(e)** HRTEM image of a twin lamella, which has a step on its lower twin boundary (marked by a white box). The thickness of the twin lamella is approximately 37 nm. An enlarged image of the step before the dislocation emission is shown in **(f)**. Scale bar: 20 nm. **(f-g)** Sequential HRTEM images showing the emission of a dislocation from the step on the TB. The height of the step is two {111} planes. **(h)** The lattice shear strain determined before the emission, corresponding to **(f)**. **(i)** Quantitative shear strain analysis of the black-box regions indicated by red and blue arrows in **(h)**. The mean shear strains near the step and in the surrounding grain interior, ε_{local}^{I} and ε_{global}^{I}, are 0.040 ± 0.001 and 0.018 ± 0.002, respectively. The loading direction is indicated by double headed arrows.

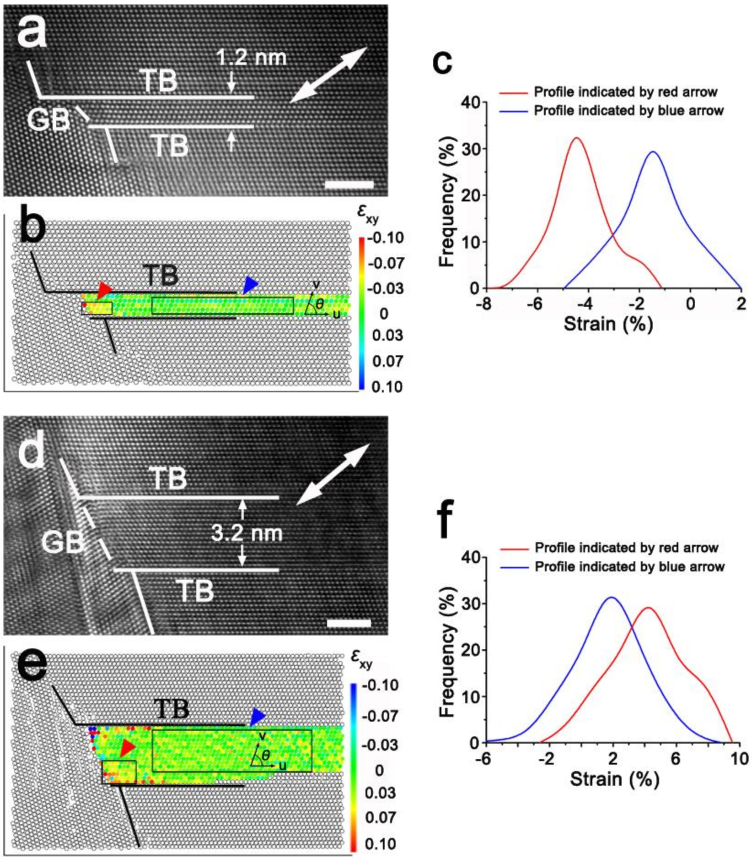

Supplementary Figure 5: Stress concentrations at junctions of TB/GBs. (a) HRTEM images of a TB/GB junction with external stress applied. The thickness of the twin lamella is approximately 1.2 nm. **(b)** Shear strain mapping corresponding to **(a)**. **(c)** Quantitative shear strain analysis of black-box regions indicated by red and blue arrows in **(b)**. The mean shear strains at the junction and in the surrounding grain interior, $\varepsilon_{\mathrm{local}}^{\mathrm{III}}$ and $\varepsilon_{\mathrm{global}}^{\mathrm{III}}$, are 0.045 ± 0.002 and 0.015 ± 0.001, respectively. **(d)** HRTEM images of a TB/GB junction with the twin lamella thickness of 3.2 nm. **(e)** Shear strain mapping corresponding to **(d)**. **(f)** Quantitative shear strain analysis of black-box regions indicated by red and blue arrows in **(e)**. The mean shear strains at the junction and in the surrounding grain interior are 0.044 ± 0.003 and 0.019 ± 0.003, respectively. The loading direction is indicated by double headed arrows. All the scale bars represent 2 nm.

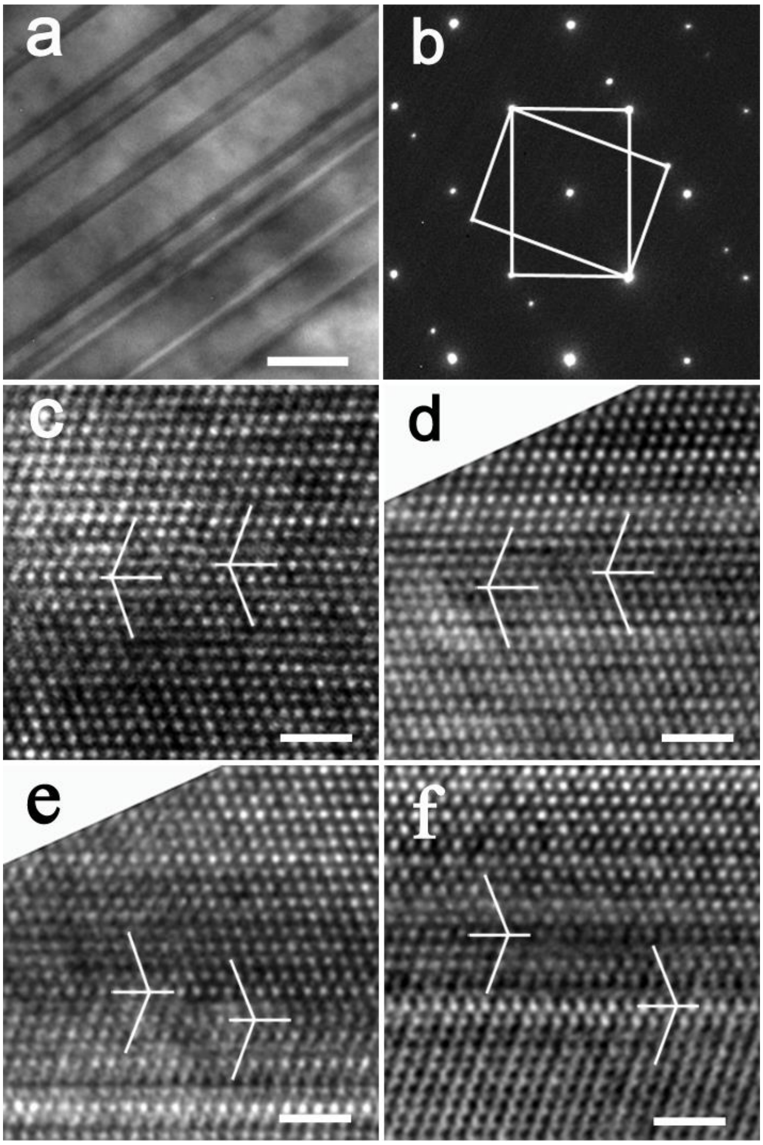

Supplementary Figure 6: Steps on twin boundaries in nanotwinned Cu. (a) The microstructure of nanotwinned Cu. Scale bar: 20 nm. **(b)** Corresponding electron diffraction pattern of **(a)**. **(c-f)** Steps with heights of 1, 1, 2 and 5 atomic layers of the (111) plane on TBs, respectively. Scale bar: 1 nm.

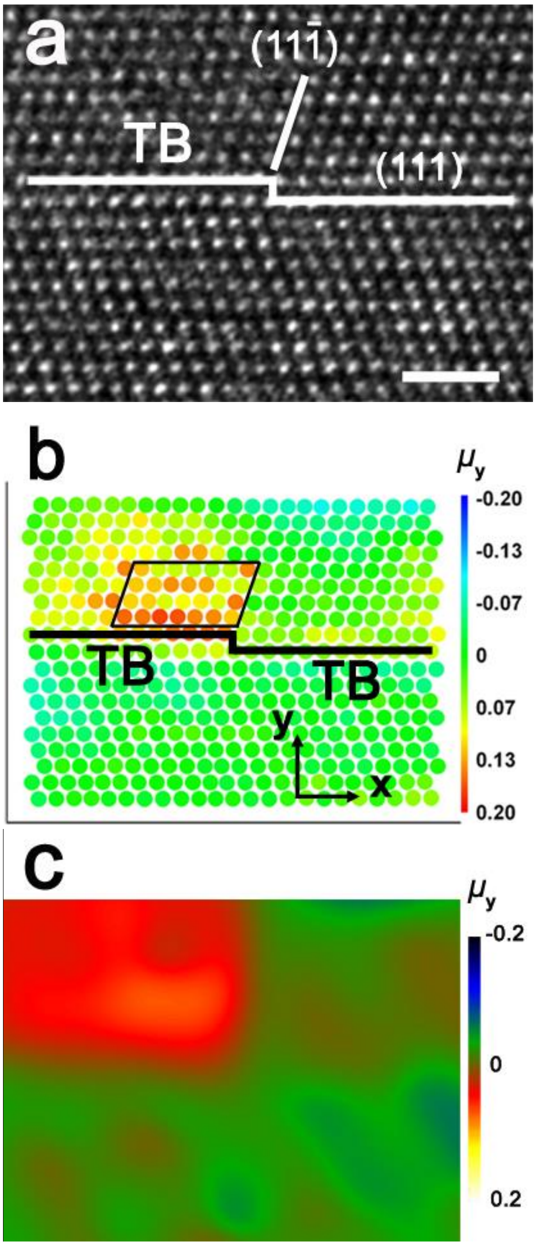

Supplementary Figure 7: Displacement of the sessile step on twin boundaries. (a) Aberration corrected high resolution transmission electron microscopy (HRTEM) image of a step on a TB. Scale bar: 1 nm. **(b)** Displacement along the **y** direction (μ_y) measured from the aberration corrected HRTEM image by the Lattice Distortion Analysis (LADIA) program. **(c)** Displacement along the **y** direction measured from the aberration corrected HRTEM image by Geometric Phase Analysis (GPA) analysis.

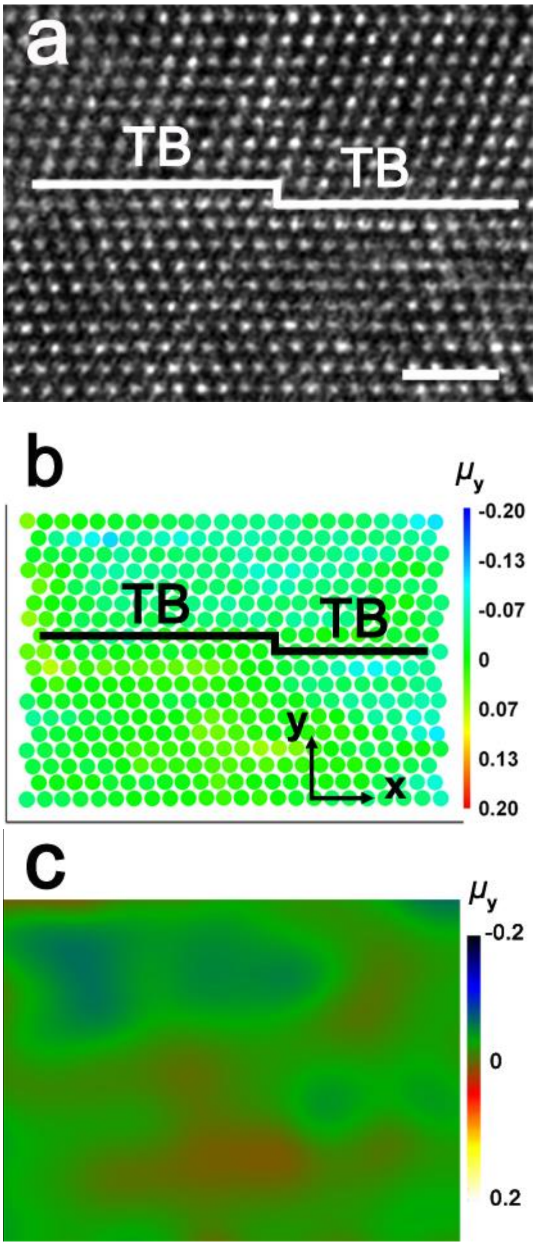

Supplementary Figure 8: Displacement of the glissile step on twin boundaries. (a) Aberration corrected HRTEM image of a step on a TB. Scale bar: 1 nm. **(b)** Displacement along the **y** direction (μ_y) measured from the aberration corrected HRTEM image by the LADIA program. **(c)** Displacement along the **y** direction measured from the aberration corrected HRTEM image by GPA analysis.

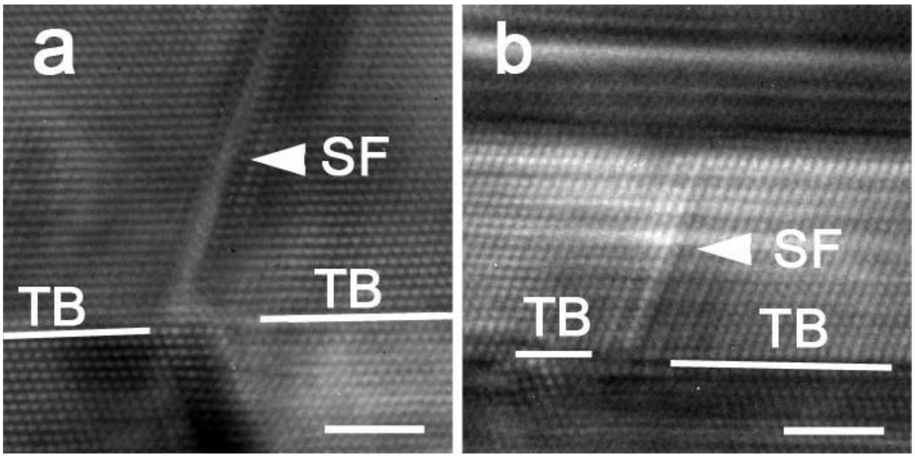

Supplementary Figure 9: Examples of the emission of type I dislocations from steps in the deformed sample with a tensile strain of approximately 0.5%. The scale bars are 2 nm.

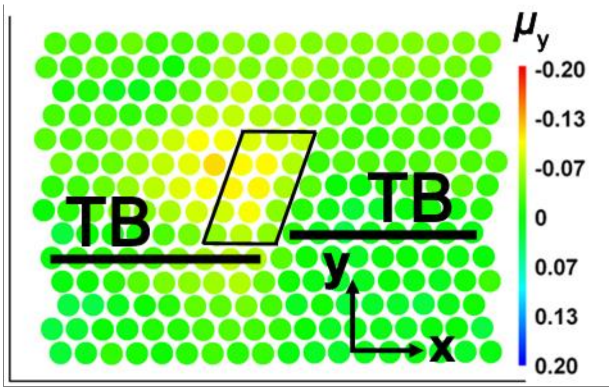

Supplementary Figure 10: Lattice displacement along the y direction in the vicinity of the step on a TB. It corresponds to the left image of Fig. 4a in the main text.

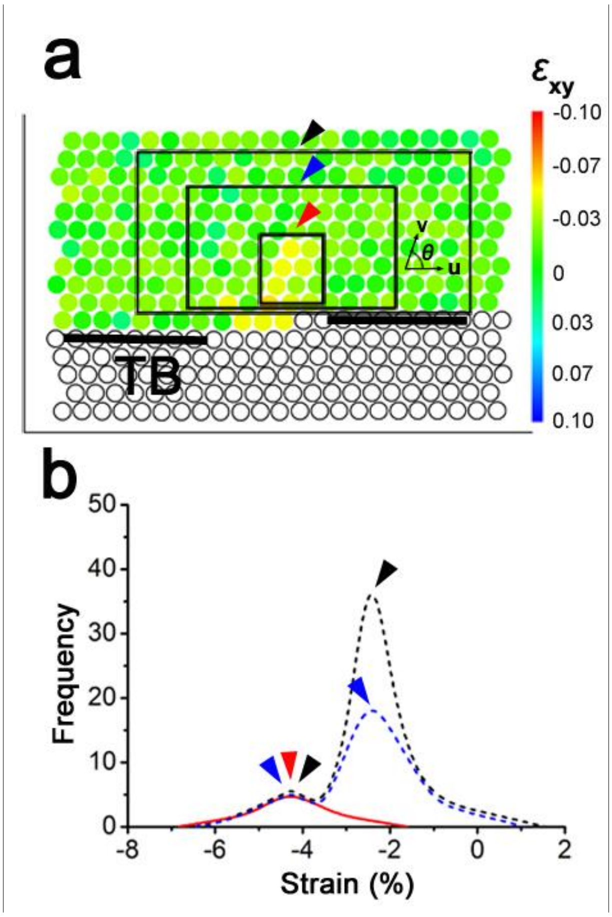

Supplementary Figure 11: Quantitative shear strain analysis of a step on the twin boundary. **(a)** The lattice shear strain ε_{xy} determined before the emission, corresponding to Fig. 4b in the main text. Different strain gauges are selected, as indicated by different black boxes. **(b)** Quantitative shear strain analysis of the black-box regions indicated by black, blue and red arrows in **(a)**.

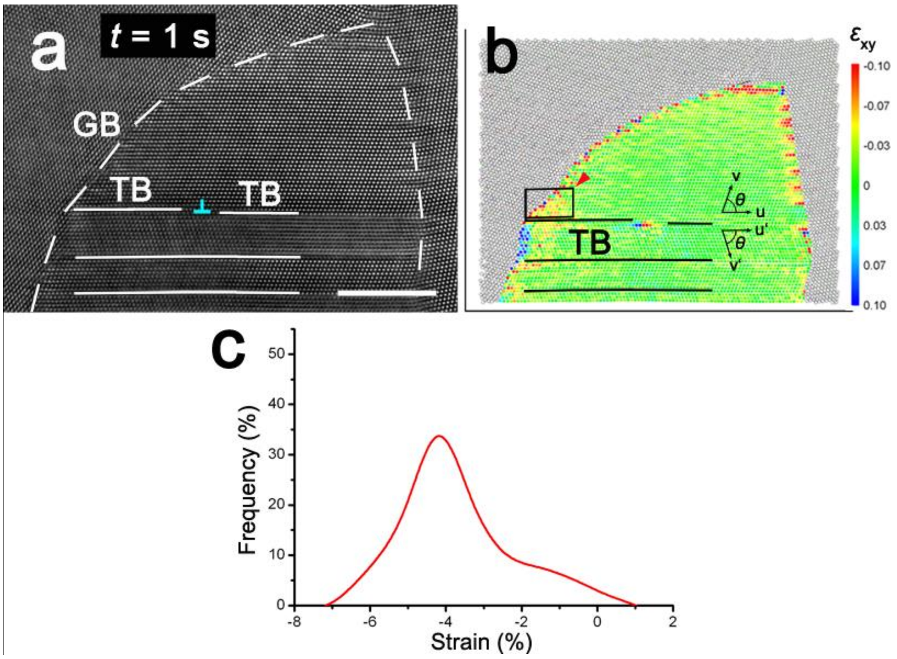

Supplementary Figure 12: Stress state of the TB/GB junction after the emission of a type III dislocation (*t*= 1 s). (a) HRTEM image of the TB/GB junction after the emission of a type III dislocation (*t*= 1 s), corresponding to the right image of Fig. 4d in the main text. The scale bar represents 5 nm. **(b)** The lattice shear strain determined after the dislocation emission, corresponding to the HRTEM image in **(a)**. **(c)** Quantitative shear strain analysis of the black-box region indicated by a red arrow in **(b)**.

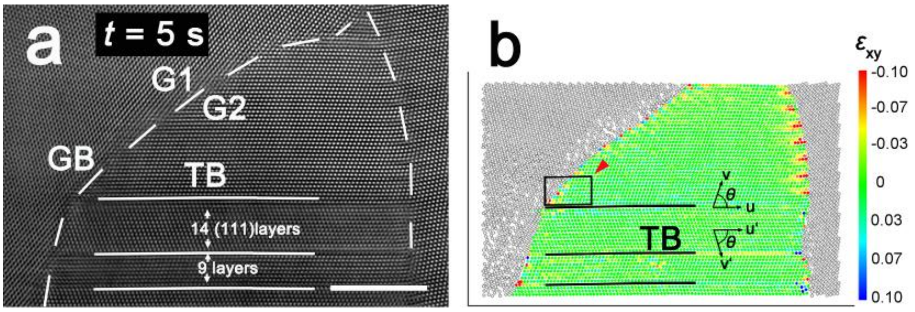

Supplementary Figure 13: Stress state of the TB/GB junction after the emission of several type III dislocations (*t*= 5 s). (a) HRTEM image of the TB/GB junction after the emission of type III dislocations (*t*= 5 s). Scale bar: 5 nm. **(b)** The lattice shear strain determined from the HRTEM image after the emission of dislocations in **(a)**.

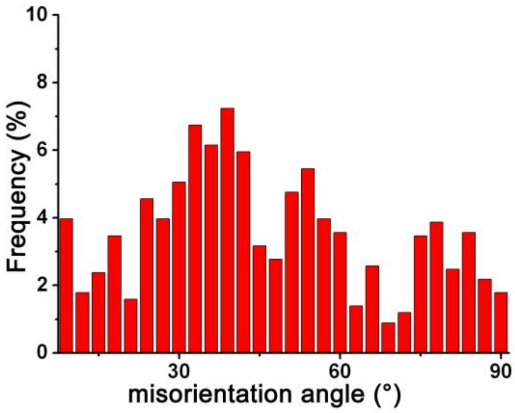

Supplementary Figure 14: The distribution of misorientation angle α between neighboring grains in the nanotwinned copper.

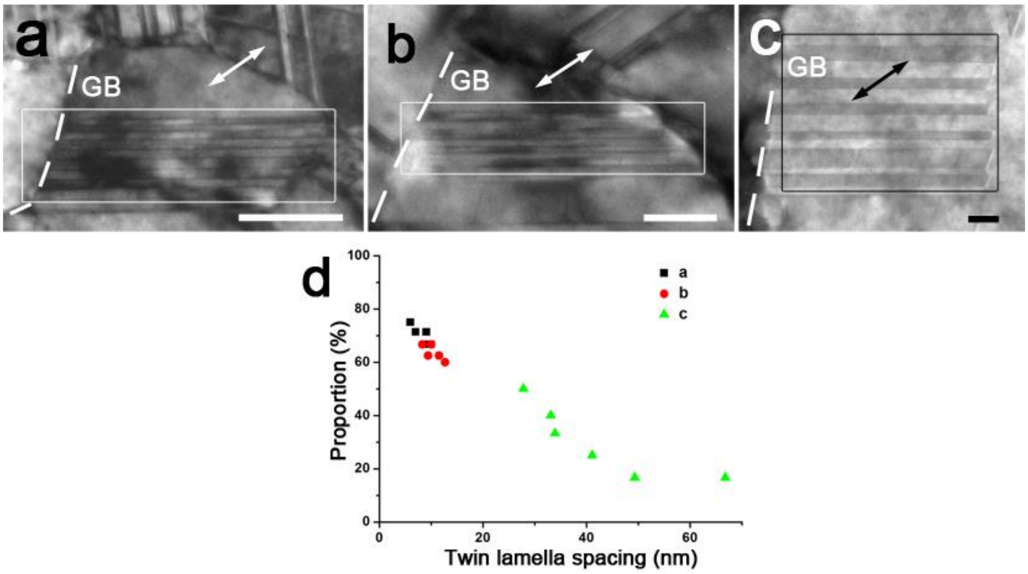

Supplementary Figure 15: Proportions of dislocations for grain boundaries with different misorientation angles or twin lamella spacings. (a-c) *In situ* bright-field images of grain boundaries with different misorientation angles but the same twin lamella spacing and grain boundaries with the same misorientation angle but different twin lamella spacings. The grain boundaries were under similar tensile direction (the angles between the tensile direction and the coherent twin boundary planes are 33-38°). All the type III dislocations were observed moving from left to right. The scale bars are 100 nm. The loading direction is indicated by double headed arrows. **(d)** Proportions of type III dislocations in rectangle boxes in **(a)**, **(b)** and **(c)**, which are indicated with black, red and green symbols, respectively.

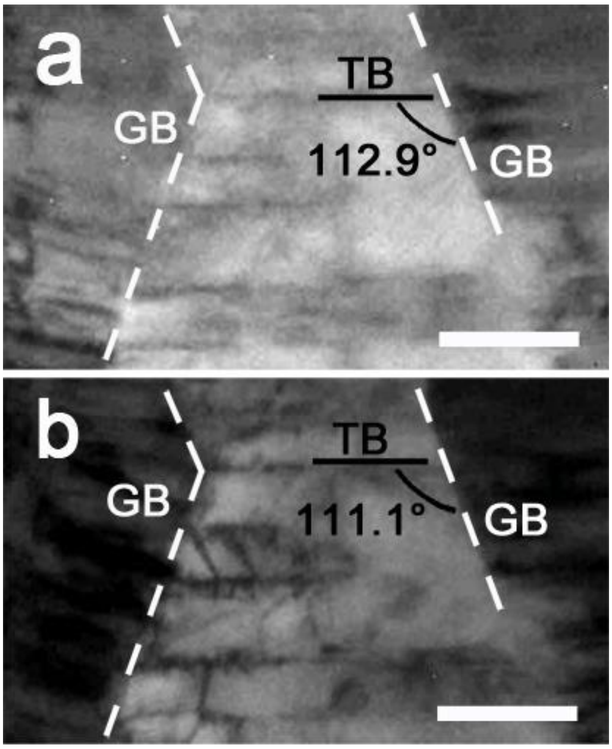

Supplementary Figure 16: The shear strain determined by the change of angle between twin boundaries and grain boundaries. Scale bars represent 100 nm.

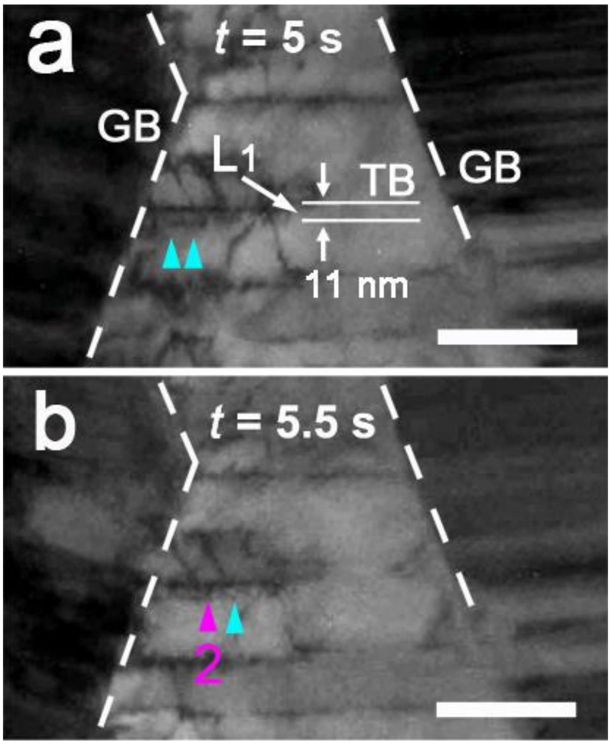

Supplementary Figure 17: Dynamic process of dislocations under a large deformation strain. (a) *t*= 5 s. **(b)** *t*= 5.5 s. Scale bars represent 100 nm.

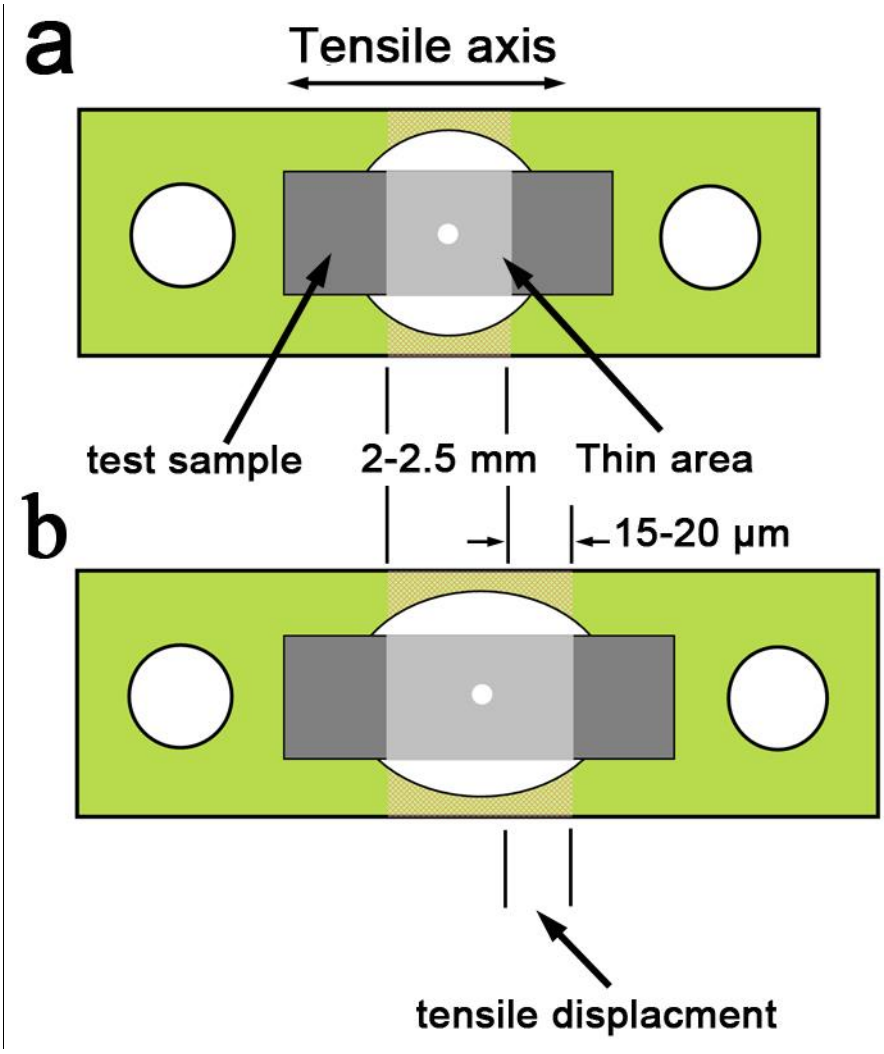

Supplementary Figure 18: Schematic illustration of a sample mounted on a Cu tensile substrate. (a) Before and **(b)** after tensile loading with a displacement of 15-20 µm.

Supplementary Tables

Supplementary Table 1: Statistical results on the proportions of three types of dislocations in nanotwinned Cu. The proportions of type I, II and III dislocations are measured from the *in situ* straining sample with an apparent strain of 1% and the post-deformed sample with a tensile strain of 0.5% investigated by transmission electron microscopy (TEM).

Status of samples	Proportion (%)		
	Type I	Type II	Type III
In situ tensile with a strain of 1%	45	6	49
Post-deformed sample with a strain of 0.5%	41	9	50

Supplementary Table 2: Determined critical shear stress for the nucleation of type I dislocations at steps with different heights on twin boundaries.

Atomic layers of step	1	1	2
Critical stress (GPa)	1.87 ± 0.14	1.69 ± 0.10	1.93 ± 0.05

Supplementary Notes

Supplementary Note 1: Step defects in as-deposited nanotwinned Cu.

Steps exist on twin boundaries (TBs) in the as-deposited nanotwinned copper. Supplementary Fig. 6a presents the microstructure of the as-deposited nanotwinned copper. The corresponding electron diffraction pattern shows typical characteristics of twins in Supplementary Fig. 6b. HRTEM images show steps on TBs in nanotwinned copper, where Supplementary Figs. 6c, 6d, 6e and 6f present steps with heights of 1, 1, 2 and 5 atomic layers of the {111} plane, respectively. Statistics on step height in as-deposited nanotwinned copper show that more than 94% of steps have a height of 1 or 2 {111} atomic layers.

Notably, aberration-corrected HRTEM images, which effectively eliminate delocalization effects from imaging[1], and quantitative lattice distortion analysis reveal two types of steps on twin boundaries in the as-deposited nanotwinned Cu (Supplementary Figs. 7 and 8). The steps shown here have the height of one (111) atomic layer. The defect configurations of these steps have apparent overall Burgers vectors of 1/6[112]*a*. To further analyze the core structures of the steps, displacement along the **y** direction is determined by the LADIA[2] and geometric phase analysis (GPA)[3] programs (Supplementary Figs. 7 and 8). Two types of core structures are clearly observed: (1) a dissociated core with obvious displacement along the **y** direction (red color in Supplementary Figs. 7b and 7c), whose motion on TBs will be inhibited by the dissociated component along the **y** direction (normal to TBs); (2) a

constricted core without considerable displacement along the **y** direction (Supplementary Fig. 8b and 8c), which is expected to be glissile on TBs. Quantitative analysis on the gauge marked by a black box (see Supplementary Fig. 7b) indicates that the mean displacement along the **y** direction in the vicinity of the step is 0.15±0.02 (the interplanar spacing of {111} is defined as the unit). The lattice displacement along the **y** direction has also been investigated by GPA, as shown in Supplementary Fig. 7c, which is consistent with the results obtained from the LADIA analysis.

This displacement along the **y** direction might be induced by the dissociation of the steps. Previous theoretical studies[4] suggest that fractions (for example, 2/9 or 4/9) of partial dislocations might exist on twin boundaries in FCC crystals. Accordingly, the displacement along the **y** direction induced by these fractions would be 2/27, 4/27 or 8/27 of the interplanar spacing of {111} planes. Here, the value of 4/27 is consistent with the above results (0.15) calculated by LADIA. Therefore, the step may dissociate into a fraction of partial dislocations on $(11\bar{1})$ and consequently introduce a lattice displacement along the **y** direction.

Supplementary Note 2: Steps with dislocation emission during the deformation process.

To further identify the effect of steps during the incipient plastic deformation, steps with dislocation emission have been investigated by post-mortem HRTEM in the deformed sample with the tensile strain of approximately 0.5%. The number of steps with dislocation emission sampled is 45. The results suggest that the steps with heights of no more than 2 {111} layers are the major sites (more than 89%) for type I dislocation nucleation. Examples of steps with the emission of type I dislocations are shown in Supplementary Fig. 9. This finding suggests that steps with multiple (i.e., more than two) {111} layers play an insignificant role in yield stress in the equiaxial-grained nanotwinned Cu.

Supplementary Note 3: Lattice displacement in the vicinity of the step on a TB, corresponding to the left image of Fig. 4a in the main text.

The step on a twin boundary corresponding to Fig. 4a has been investigated. Lattice displacement along the **y** direction was measured by the LADIA program, as shown in Supplementary Fig. 10. A large lattice displacement in the area marked by a black box suggests that the step is dissociated on the {111} plane inclined to the TB. Based on the previous discussion in the Supplementary Note 1, this step has a dissociated core

and is thus inhibited from moving on the TB. Quantitative analysis on a gauge marked by the black box in Supplementary Fig. 10 indicates that the mean displacement along the **y** direction in the vicinity of the step is 0.08±0.02 (the interplanar spacing of {111} is defined as the unit). This is also consistent with the Supplementary Note 1 in that the displacement induced by this fraction of dislocation is possibly 2/27 of the interplanar spacing of {111} planes.

Supplementary Note 4: The validity of the selected strain gauge around the step.

Supplementary Fig. 11 demonstrates different sizes of the strain gauge covering areas of the matrix and steps for quantitative analysis. These gauges are marked by black boxes (indicated by black and blue arrows) in Supplementary Fig. 11a. The results of quantitative strain analysis have been plotted in Supplementary Fig. 11b, as indicated by the black and blue traces. For example, the black trace demonstrates that there are two peaks of the strain value (denoted by black arrows): the right peak indicates the strain of the matrix and the left one indicates the existence of stress concentration. The left peak for the stress concentration in the blue trace is almost the same as that in the black trace. This finding suggests that different sizes of the strain gauge will not significantly influence the presentation of stress concentration around the steps on

TBs in Supplementary Fig. 11a. In addition, the result of the strain analysis on the proximity of the step has also been plotted in Supplementary Fig. 11b. The strain gauge marked by a black box (indicated by a red arrow in Supplementary Fig. 11a and corresponds to the result in Fig. 4b) gives a strain distribution, presented as the red trace in Supplementary Fig. 11b. The strain distribution is almost the same as the left peaks of the strain from the large areas. Therefore, the selected strain gauge (marked by a black box and indicated by a red arrow in Fig. 4b) can represent the strain distribution of the stress concentration at the steps on TBs faithfully.

Supplementary Note 5: The influence of step heights on the critical stress required for the emission of type I dislocations.

The critical stress has been measured for the nucleation of type I dislocations at steps with heights of 1 or 2 atomic layers (Supplementary Table 2). These results show that the critical stress values are roughly the same, which suggests that the stress required for dislocation emission would be constant at steps with one or two atomic layers. In addition, if we consider the dislocation emission from the steps as a process of dislocation dissociation, then the stress required for the dislocation nucleation should only be related to the Burgers vectors at the step and of the emitted dislocation and thus would be independent of the step height. Here, the critical shear stress required

for dislocation emission can be estimated based on the assumption that the shear stress for dislocation dissociation corresponds to the attractive force between two parallel straight dislocations when the distance between them is equal to the core width of the initial dislocation. In this way, the shear stress is sufficient to dissociate the initial dislocation into two dislocations[5-6]. The radial force between two parallel dislocations can be given as

$$F = \frac{G}{2\pi d}[(b_1 * \xi) \times (b_2 * \xi)] + \frac{G}{2\pi(1-\upsilon)d}[(b_1 \times \xi) * (b_2 \times \xi)] \qquad (1)$$

where d is the distance between two dislocations, G is the shear modulus, ξ is the dislocation line vector, v is the Poisson ratio, and b_1 and b_2 are Burgers vectors of the two parallel dislocations[5-6]. Therefore, the shear stress required for the dislocation emission at steps should be independent of the step height, if the Burgers vectors at the steps b_1 and of the emitted dislocation b_2 are constant for those steps. Combining the above results with the fact that more than 94% of steps have a height of no more than 2 atomic layers, it can be assumed that dislocation emissions from steps with one or two atomic layers are dominant variety of slips across twin lamellae during tensile deformation.

Supplementary Note 6: The stress state after the emission of type III dislocations.

Supplementary Fig. 12a (corresponding to the right image of Fig. 4d in main text) shows a HRTEM image taken after the emission of a type III dislocation. The corresponding strain distribution is shown in Supplementary Fig. 12b. The quantitative strain profile (Supplementary Fig. 12c) from a strain gauge marked by a black box (indicated by a red arrow) in Supplementary Fig. 12b indicates that the mean shear strain near the twin boundary/grain boundary (TB/GB) junction is 0.042 ± 0.002, which corresponds to a local shear stress of 2.02 ± 0.10 GPa.

Under the continuous tensile loading, several dislocations were observed being sequentially emitted from TB/GB junctions, as shown in Fig. 4d and Supplementary Fig. 13a. This phenomenon is confirmed by the thickness of the twin lamella, as it was eleven {111} layers in the left image of Fig. 4d and fourteen {111} layers in Supplementary Fig. 13a, which suggests that three type III dislocations passed along the upper TB. In the meantime, no dislocation activity was observed on the lower twin boundary (the thickness of the lower twin lamella remains at 9 {111} layers during the loading process). Thus, the lower TB can be used as a reference for the change of the upper twin boundary.

The time between the left (t = 0 s) and right (t = 1 s) images of Fig. 4d is 1 s, which is sufficient for the stress concentration to accumulate again at the TB/GB junction after

the first type III dislocation emitted from the TB/GB junction. Therefore, the local shear strain before the next dislocation emission (2.0 GPa, the right image of Fig. 4d) could be comparable to the strain before the first dislocation's emission (2.2 GPa, the left image of Fig. 4d) from the TB/GB junction.

Supplementary Fig. 13a (*t*= 5 s) corresponds to 5 s after the left image of Fig. 4d, and the stress concentration was fully relaxed at the TB/GB junction there. The corresponding shear strain is shown in Supplementary Fig. 13b. No significant stress concentration is observed at the junction of the TB/GB (black box indicated by a red arrow). This suggests that the stress concentration was fully relaxed at the TB/GB junction after 5 s and the emission of several type III dislocations.

Supplementary Note 7: The influence of grain boundary structure and twin lamella spacing on the dislocation activities.

The stress required for dislocation nucleation at GBs has been investigated by Molecular Dynamic (MD) and quasicontinuum simulations on bi-crystals with different misorientation angles[7]. According to the reported results, the fluctuation of the stress required for dislocation nucleation from the GB is relatively small in the range of 35°<θ<80° or 115°<θ<145°, where θ is the misorientation angle between two grains. Considering that a quadratic rotation axis exists along the [110] direction in a face-centered cubic crystal, the misorientation angle can be determined in the range of

0°<θ<90°. Hence, the stress required for dislocation nucleation should not vary significantly in the range of 35°<θ<65°. We have conducted a statistical analysis on the distribution of misorientation angles between grains in the as-deposited nanotwinned copper specimen, as shown in Supplementary Fig. 14. The misorientation angles mainly lie between 25° and 65°, and 80% of them are between 35° and 65°. Combining the above result with MD simulations, it is reasonable to assume that the stress required for the dislocation nucleation at the junction of the TB/GB could be roughly constant for the grains in our equiaxial-grained nanotwinned Cu specimens. Notably, θ=39°, corresponding to ∑9 GB, has the highest proportion among all the GBs in the specimen. The statistical results of the stress concentration factor at the twin boundary/grain boundary junctions are all determined at the near ∑9 GBs during the experiment; hence, the determined stress concentration factors are representative for grain boundaries in the specimen.

To further investigate the influence of the grain boundary structure and twin lamella spacing on the dislocation activities, the proportions of two types of dislocations have been examined for grain boundaries with different misorientation angles but the same twin lamella spacing and also for grain boundaries with the same misorientation angle but different twin lamella spacings, as shown in the example in Supplementary Fig. 15. We verify that all the grain boundaries are roughly under the same tensile direction by checking the angle between the tensile direction and the coherent twin boundary planes (33°-38°). This ensures that they are under similar stress conditions

during the experiment. All the type III dislocations were observed moving from left to right. The TB/GB junctions where dislocations nucleated are located at the left side of the grains. Supplementary Figs. 15a and 15b show grain boundaries with different misorientation angles (30° and 40°, respectively) but the same twin lamella spacing (approximately 10 nm). As shown in Supplementary Fig. 15d, the proportions of type III dislocations are roughly the same (approximately 60-70%, indicated by black and red symbols). Supplementary Figs. 15b and 15c show grain boundaries with the same misorientation angle (40°) but different twin lamella spacings (10 nm and 25-70 nm). As shown in Supplementary Fig. 15d, the proportions of type III dislocations are different, 60-70% and 20-50% (indicated by red and green symbols). This finding suggests that the twin lamella spacing, rather than the grain boundary structure (presented as grain boundary misorientation angle here), plays a crucial role in the dislocation nucleation.

Supplementary Note 8: Determination of the shear strain during the large strain deformation.

As shown in Supplementary Fig. 16a (corresponding to Fig. 6a), the included angle between the twin boundary and grain boundary is 112.9°. After the loading of external stress, the included angle becomes 111.1° (Supplementary Fig. 16b, corresponding to Fig. 6c). Thus, the shear strain can be estimated as

$$\varepsilon = (111.1^\circ - 112.9^\circ) * \pi / 180 = 0.03\,. \qquad (2)$$

In addition, the tensile displacement is about 85 μm at the time of capturing Fig. 6c, and thus the apparent strain is roughly estimated by the ratio of the tensile displacement to the length of the deformed area as about 4%. This agrees well with the above result of 3% shear strain at the time of Fig. 6c.

Supplementary Note 9: The mechanism of deformation with a large strain in nanotwinned Cu.

As described above, for $\lambda<\lambda_c$ in the incipient plastic deformation, dominant type III dislocations are emitted from TB/GB junctions and slip along the coherent twin boundaries, as shown in Fig. 6b. After the emission of abundant type III dislocations, as shown in the Fig. 6c, type I dislocations (like dislocations 1 and 2) were also observed to be emitted. Nevertheless, due to the limitation of the time resolution (0.5 second per frame) and spatial resolution of the *in situ* bright-field TEM observation, the detailed process of the emission of type I dislocations cannot be resolved at a large strain. One possibility is that type I dislocations are emitted from TB/GB junctions (e.g., dislocation 1 in Fig. 6c) or the inherent steps on TBs (e.g., dislocation 2). As shown in Supplementary Figs. 17a and 17b (bright-field images taken before (*t*= 5 s) and after (*t*= 5.5 s) the emission of dislocation 2), type III dislocations (indicated by blue arrows) were still moving, although other type III dislocations started to accumulate at the left side of the grain. These results suggest that dislocation 2 is likely emitted from an inherent step rather than a (still moving) type III dislocation. In

addition, the stress will increase with the pile-up of type III dislocations and may be sufficient to trigger the emission of a type I dislocation. Nevertheless, as stated, the possibility of the emission of type I dislocations by the dissociation of piled-up type III dislocations cannot be completely excluded due to the above mentioned limits of *in situ* bright-field TEM observation. Notably, this emission of type I dislocations is different than the nucleation of type I dislocations (e.g., as shown in Supplementary Movie 4) that has often been observed without prior activity of type III dislocations in the incipient deformation.

Supplementary References

1 Haider, M. *et al.* Electron microscopy image enhanced. *Nature* **392**, 768-769 (1998).

2 Du, K., Rau, Y., Jin-Phillipp, N. Y. & Phillipp, F. Lattice distortion analysis directly from high resolution transmission electron microscopy images - the LADIA program package. *J. Mater. Sci. Technol.* **18**, 135-138 (2002).

3 Hÿtch, M. J., Snoeck, E. & Kilaas, R. Quantitative measurement of displacement and strain fields from HREM micrographs. *Ultramicroscopy* **74**, 131-146 (1998).

4 Zhu, Y. T. *et al.* Dislocation–twin interactions in nanocrystalline fcc metals. *Acta Mater.* **59**, 812-821 (2011).

5 Hirth, J. P. & Lothe, J. *Theory of Dislocations*. (McGraw-Hill 1968).

6 Zhu, Y. T. *et al.* Twinning partial multiplication at grain boundary in nanocrystalline fcc metals. *Appl. Phys. Lett.* **95**, 031909 (2009).

7 Tschopp, M. A., Tucker, G. J. & McDowell, D. L. Structure and free volume of <1 1 0> symmetric tilt grain boundaries with the E structural unit. *Acta Mater.* **55**, 3959-3969 (2007).
